# Do Endometrial Movements Affect The Achievement of Pregnancy during Intrauterine Insemination?

**DOI:** 10.22074/ijfs.2015.4180

**Published:** 2015-02-07

**Authors:** Ari Kim, Ji Young Lee, Yong Il Ji, Hae Hyeog Lee, Eun Sil Lee, Heung Yeol Kim, Young Lim Oh

**Affiliations:** 1 Department of Obstetrics and Gynecology, Institute of Wonkwang Medical Science, College of Medicine, Wonkwang University, Iksan, Korea; 2 Department of Obstetrics and Gynecology, College of Medicine, Konkuk University, Seoul, Korea; 3 Department of Obstetrics and Gynecology, College of Medicine, Inje University, Busan, Korea; 4 Department of Obstetrics and Gynecology, College of Medicine, Soon Chun Hyang University, Seoul, Korea; 5 Department of Obstetrics and Gynecology, College of Medicine, Kosin University, Busan, Korea

**Keywords:** Pregnancy Outcome, Endometrium, Endometrial Cycle, Insemination, Clomiphene Citrate

## Abstract

**Background:**

This study was aimed to assess the effect of endometrial movements on pregnancy achievement in intrauterine insemination (IUI) cycles.

**Materials and Methods:**

The population of this observational study was composed of unexplained infertility couples undergoing first-time IUI with clomiphene citrate between September 2010 and October 2011. Not only endometrial movements, but also thickness, volume, pattern, and echogenic change of endometrium were analyzed prospectively in prediction of pregnancy.

**Results:**

The total number of 241 cycles of IUI with 49 intrauterine pregnancies (20.3%) was analyzed. Pregnancy was not related to endometrial thickness and endometrial volume, but significantly related to endometrial movements associated with the number of contraction, strong movement, cervicofundal direction, and hyperechoic change (p<0.05). Pregnant group showed higher cervicofundal movement rate (89.8 vs. 75.5%).

**Conclusion:**

For IUI cycles stimulated by clomiphene citrate in unexplained infertility women, endometrial movements on the day of IUI could be a predictor of pregnancy.

## Introduction

As a complex reproductive organ, the uterus plays an active role in the early stages of pregnancy by providing a site for implantation and promoting the transport of spermatozoa (1-3). The directed transport of spermatozoa and a high fundal implantation rate of embryo result, at least in part, from endometrial movements (1, 2, 4, 5). Such movements are thought to be controlled by hormonal changes initiated by ovaries (4).

Endometrial movements in the nonpregnant uterus have been studied using invasive and noninvasive methods. Endometrial activity was initially documented using intrauterine pressure recordings of the intensity, amplitude, and frequency of myometrial contraction (6). Ultrasound technology enables precise noninvasive imaging of endometrial movements. The inner part of the myometrium has been revealed to generate contractions and endometrial wave-like movements (7).

Although several studies have assessed endometrial movements and pregnancy rates resulting from *in vitro* fertilization (IVF) and have concluded in negative correlation between the frequency of movements and pregnancy outcomes (7-9), few have examined the relationship between endometrial movements and pregnancy achievement after intrauterine insemination (IUI). In IVF cycles, implantation is a key determinant of pregnancy, whereas both processes of spermatozoa transport and implantation occur after IUI. Thus, the influence of endometrial movements on the achievement of pregnancy may differ in IUI compared to IVF cycles or may even helpful for pregnancy in IUI cycles. In this reason, we departmentalized those movements and analyzed to assess whether endometrial movements were predictive of pregnancy rates in patients with unexplained infertility who underwent ovarian stimulation with clomiphene citrate followed by IUI.

## Materials and Methods

### Patient recruitment

The population of this observational study was composed of couples who had been considered infertile for at least 1 year, who did not have any known infertility factors, and who planned to undergo first-time IUI with clomiphene citrate (CC) at the Fertility Centers of 3 University Hospitals (Wonkwang, Kosin, Soon Chun Hyang), Korea, between September 2010 and October 2011. A total of 243 infertile couples with 243 cycles were enrolled in study. Before each course of treatment, a medical history was obtained from each patient, physical examinations were performed and the following tests were conducted to determine the cause of infertility: diagnostic vaginal ultrasound, hysterosalgography, serum hormone assays on the third day of the menstrual cycle [including measurements of follicle-stimulating hormone (FSH), luteinizing hormone (LH), estradiol, prolactin, and testosterone], and semen analysis. Patients with endometrioma, ovarian cysts, tubal obstruction, severe adenomyosis, uterine fibroids, endometrial polyps, severe pelvic adhesion, uterine anomaly, ovulatory dysfunction, or male factor infertility or subfertility were excluded from the study. After routine examinations, couples who have been diagnosed for the first time with unexplained infertility and had no history of urologic or gynecologic operations were included in the study. No therapeutic interventions except routine procedures were performed on the patients. The study protocol was approved by the Institutional Review Board of Kosin University, and each woman provided verbal informed consent.

### Ovarian stimulation and IUI procedures

Ovulation induction began on the third day of the menstrual cycle and lasted for 5 days, with 100 mg of CC administered after using conventional transvaginal ultrasound to confirm the absence of pathologic conditions. Transvaginal ultrasound was applied on days 10 to12 of the menstrual cycle to monitor follicular and endometrial development, and the ultrasound was repeated every 1 to 2 days to determine the proper time of human chorionic gonadotropin (hCG) (Pregnyl®; Organon, Netherland) administration. When the size of leading follicle reached ≥18 mm in diameter, 10,000 IU hCG was administered.

After 36 hours of hCG administration, a single IUI was performed. A semen specimen was obtained by masturbation and collected in a sterile container. The specimen was processed with mixed media of Ham’s F-10, (Gibco Invitrogen, Carlsbad, CA), penicillin/streptomycin (Gibco Invitrogen, Carlsbad, CA) and sodium polyanethol sulfonate (SPS). During the wash cycles and a 30-minute swim-up period, the sperm specimen was centrifuged twice at 1,500 rpm for 5 minutes each. Semen quality was determined according to values reported by the World Health Organization (WHO) (10). In basic semen analysis, we estimated pH, volume, sperm count, sperm motility, sperm morphology, white blood cell (WBC) count, viscosity, and agglutination of the obtained semen. After semen processing, the semen parameters were re-estimated, and the sperm motility was over 90% in all cases. The cervix was exposed using bivalve speculum and cervical mucus was cleaned with a cotton dressing. The fully processed sperm specimen was drawn into an artificial insemination catheter (Wallace®, Smiths Medical International Ltd., Hythe, Kent, United Kingdom), and then instilled into the upper portion of the uterine cavity. After insemination procedure, the patient rested in a supine position for about 15 minutes. All of patients had luteal support with two times of hCG injection after IUI. Qualitative hCG urine tests were performed 14 days after insemination to determine if a clinical pregnancy had been established. Clinical pregnancy was confirmed via the detection of gestational sac via transvaginal ultrasound scanning.

### Ultrasound evaluation

Along with routine serial ultrasound examinations to check endometrium and follicles on the day of IUI, additional ultrasound evaluations (ACCUVIX XQ, Medison, Seoul, Korea) with high resolution mode were performed to assess various characteristics of the endometrium. The results of the adjunctive ultrasound did not affect subsequent clinical management procedures. The number of remained follicles with diameters greater than 16 mm was considered for potential rupture.

Endometrial movement was observed and recorded on compact disk (CD) for 3 minutes. The movies were reviewed in fast-forward mode and normal-forward mode to be analyzed with normal and high speed by 3 observers to avoid interobserver variation, with regards to wave frequency, predominant direction, and degree of endometrial movement. Wave frequency was defined as the number of waves during a 3 minutes period, divided by three, regardless of the longitudinal direction of each wave. There was no interobser and intraobserver variation. The predominant direction of endometrial movement was determined by assessing the peristaltic activity of the endometrial border. Because we focused on the longitudinal waves rather than transverse or weak focal activities, the dominant direction was classified as either fundocervical or cervicofundal. All patients showed their own longitudinal wave directions. The degree of endometrial movement as rated on a three-point scale, with 0 indicating weak movement, 1 indicating moderate movement and 2 indicating vigorous movement. Weak movement was defined subendometrial wave-like activity without endometrial conduction or movement. Vigorous movement was defined prominent endometrial stream with distinct subendometrial wave-like activity.

Endometrial thickness was measured as the maximum distance between the myometrial and endometrial interfaces in the midsagittal plane of the uterus, after the wave-like appearance of the endometrial border has disappeared.

Endometrial volume was calculated using virtual organ computer-aided analysis (VOCAL) software built into the ultrasound machine. The areas of interest included the endometrium and subendometrial regions within 5 mm of the echogenic endometrial borders. Three-dimensional volume data were obtained using the automatic sweep function, with an angle of 90˚ to include a complete uterine volume that encompassed the entire subendometrium. The resulting multiplanar display was examined to ensure that the area of interest had been captured in its entirety. The manual mode of the VOCAL Contour Editor was used to include the entire volume of the endometrium using a 15˚ rotation step. Twelve endometrial slices were obtained by outlining the endometrium at the myoendometrial junction from fundus to the internal os (11).

Two (i.e., trilaminar and nontrilaminar) patterns of the endometrium were classified. A trilaminar pattern was defined as a hypoechoic layer with a central echogenic line or an isoechoic layer with a central hyperechoic line. A nontrilaminar pattern was described as a single homogeneous endometrium.

**Fig 1:**
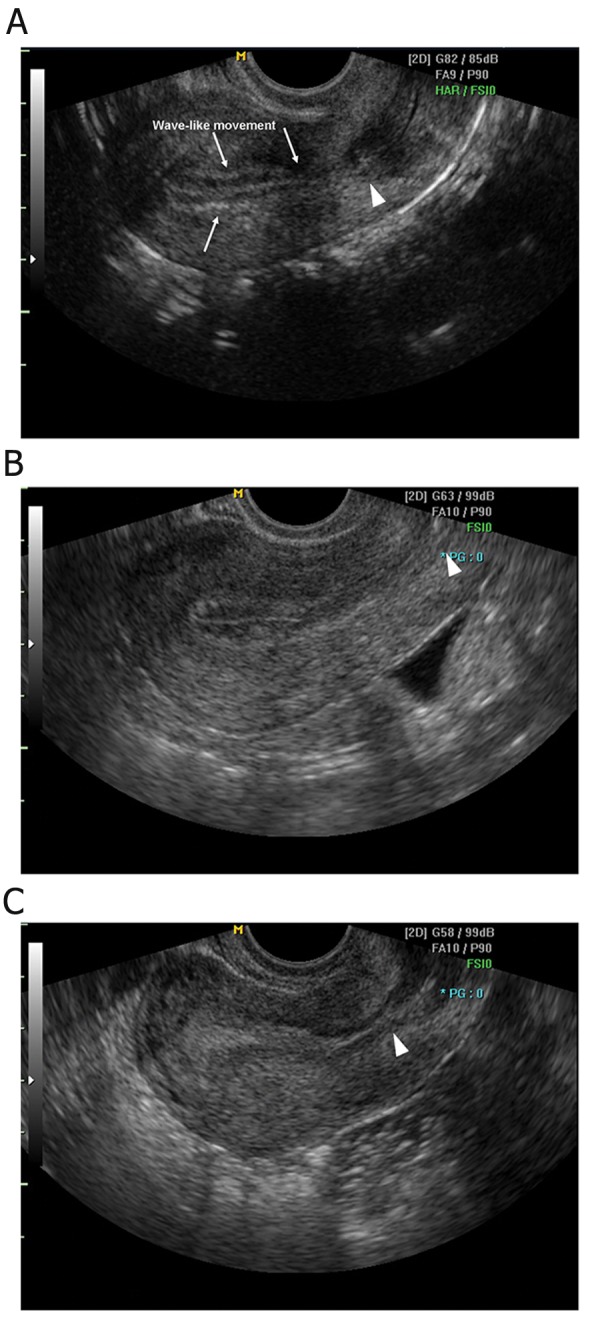
Ultrasound images of endometrium. Echogenisity was decided by the comparison of myomertial echo. Homogeneously hypoechoic endometrium (A) is defined as a marginal hyperechoic line with a prominent hypoechoic inner layer, in which endometrial wave-like movements are observed. Inhomogeneous endometrium (B) shows an endometrium with a hyperechoic margin and a mottled inner layer, and homogeneously hyperechoic endometrium (C) exhibits an inner layer as hyperechoic as a marginal line. Internal os (arrowheads) can be identified.

Hyperechoic changes with the endometrium were scored according to the degree of echoicity, with 0 corresponding with homogeneously hypoechoic findings, 1 corresponding with inhomogeneous findings, and 2 corresponding with homogeneously hyperechoic findings (Fig 1). Echogenisity was decided by the comparison of myomertial echo. A homogeneously hypoechoic endometrium was defined as a marginal hyperechoic line with a prominent hypoechoic inner layer. An inhomogeneous endometrium was defined as an endometrium with a hyperechoic margin and a mottled inner layer. A homogeneously hyperechoic endometrium was defined by an inner layer as a marginal hyperechoic line.

### Statistical analysis

Results are presented as mean and standard deviation values. Normal distribution was defined using the Kolmogorov-Smirnov test. To assess continuous variables, comparisons between two groups were performed using a Student’s t test. Statistic tests of non-continuous and categorical variables were carried out using the χ2 test and Fisher’s exact test. Data was considered statistically significant if p<0.05. Logistic regression analysis was performed to test for correlations between clinical or ultrasonographic variables and the occurrence of pregnancy in subgroup of women with endometrial trilaminar pattern. Odds ratios (ORs) and 95% confidence intervals (95% CIs) were estimated separately for each variable. Confidence intervals exclusive of unity were considered to be statistically significant. The statistical analyses were performed using the Statistical Package for Social Sciences (SPSS; SPSS Inc., Chicago, USA).

## Results

Excluding two cases of tubal pregnancy, a total of 241 cycles and 49 cases of intrauterine pregnancies (21.0%) were examined. The study population was ranged in age from 23 to 44 years. Comparisons of clinical in the nonpregnant and pregnant groups are summarized in table 1. There were no significant differences between the nonpregnant and pregnant groups with regards to mean IUI day, interval from hCG injection to IUI, numbers of preovulatory or postovulatory follicles, or uterine version. However, the mean female age was significantly lower in the pregnant group.

The endometrial characteristics of the pregnant and nonpregnant groups are summarized in table 2. Pregnancy was not related to endometrial thickness and endometrial volume, but was significantly related to endometrial movement, trilaminar pattern, and hyperechoic change. Although there was no difference in the mean number of endometrial movements in the nonpregnant and pregnant groups, more than four movements per minute were significantly related to pregnancy. The major direction of endometrial movement was cervicofundal (82.6%) and the pregnant group exhibited a higher rate of cervicofundal movement (89.8 vs. 75.5%). The mean number of contractions was 3.94 ± 1.75 in the cervicofundal direction, and 2.23 ± 1.35 in the fundocervical direction. Statistically, cervicofundal movement was related to pregnancy. In terms of the degree of endometrial movement, vigorous movement was significantly indistinct in pregnant group. Tubal pregnancies were recorded in women who exhibited vigorous endometrial movements of four and five contractions per minute, both in the cervicofundal direction.

As a static parameter, trilaminar patterns of the endometrium were significantly related to pregnancy. No pregnancies occurred in 21 cases of nontrilaminar pattern. In terms of hyperechoic changes, a significantly lower pregnancy rate of 13.5% was noted in women with homogeneous hyperechoic endometrium. A subgroup analysis of endometrial echogenicity revealed that the highest pregnancy rate occurred in women with inhomogeneous endometrium (29.3%), while women with homogeneously hyperechoic endometrium exhibited the lowest pregnancy rate (7.4%).

It is unclear what role trilaminar patterns played in the results of our analysis, as no pregnancy occurred in nontrilaminar cases. Thus, logistic regression analysis was performed using the significant variables listed in tables 1 and 2. Table 3 showed that the hyperechoic endometrium group was compared with the hypoechoic and inhomogeneous endometrium groups. The higher number of endometrial movements and vigorous subendometrial contractions were also significant for pregnancy. On the other hands, patients’ age did not showed the significance for the occurrence of pregnancy when the endometrium had the trilaminar. There was no significant difference with regards to the direction of endometrial movement in the analysis restricted to the trilaminar endometrium, unlike the outcome of table 2.

**Table 1:** Comparison of clinical characteristics between the pregnant and nonpregnant groups


Characteristics	Nonpregnant group	Pregnant group	p
	(n=192)	(n=49)	

**Female age**	32.86 ± 3.56	31.43 ± 3.06	0.010^a^
**Duration of infertility**	1.75 ± 9.21	1.55 ± 7.46	0.125^a^
**IUI day**	15.74 ± 3.17	15.14 ± 2.42	0.216^a^
**Interval from hCG injection to IUI (hours)**	37.25 ± 5.95	36.98 ± 2.63	0.757^a^
**Number of follicles**	3.16 ± 2.25	3.76 ± 3.00	0.123^a^
**Number of preovulatory follicles (≥16 mm)**	1.65 ± 2.15	2.20 ± 2.99	0.143^a^
**Number of postovulatory follicles (corpus luteum)**	1.52 ± 1.29	1.55 ± 1.08	0.875^a^
**Uterine version**			
**Anteversion**	154 (80.2%)	37 (75.5%)	0.469^b^
**Retroversion**	38 (19.8%)	12 (24.5%)	


Mean values ± SD or percentage and number of cases are given in parentheses. IUI; Intrauterine insemination, hCG; Human chorionic gonadotropin, ^a^; Student’s t test and ^b^; χ^2^ test.

**Table 2:** Comparison of endometrial characteristics evaluated by ultrasound between pregnant and nonpregnant groups


Characteristics	Nonpregnant group(n=192)	Pregnant group(n=49)	p

**Endometrial thickness**	10.85 ± 2.69	11.44 ± 2.31	0.719^a^
**Endometrial volume**	5.16 ± 2.61	5.84 ± 2.68	0.108^a^
**Endometrial movement**			
**Number of contractions**	3.46 ± 1.89	4.02 ± 1.44	0.053^a^
**≤4 times/minute**	110 (57.3%)	19(38.8%)	0.020^b^
**>4 times/minute**	82 (42.7%)	30(61.2%)	
**Direction**			
**Cervicofundal**	145 (75.5%)	44(89.8%)	0.030^b^
**Fundocervical**	47 (24.5%)	5 (10.2%)	
**Degree of endometrial movements**			
**Weak**	81 (42.2%)	13(26.5%)	0.017^c^
**Moderate**	81 (42.2%)	32(65.3%)	
**Vigorous**	30 (15.6%)	4 (8.2%)	
**Trilaminar pattern**			0.010^c^
**Trilaminar**	171 (89.1%)	49(100%)	
**Nontrilaminar**	21 (10.9%)	0 (0%)	
**Hyperechoic change**			0.001^b^
**Homogeneously hypoechoic**	59 (30.7%)	19(38.8%)	
**Inhomogeneous**	58 (30.2%)	24(50.0%)	
**Homogeneously hyperechoic**	75 (39.1%)	6 (12.2%)	


Mean values ± SD or percentage and number of cases are given in parentheses. ^a^; Student’s t test, ^b^; χ^2^ test and ^c^; Fisher’s Exact test.

**Table 3:** Factors associated with the occurrence of pregnancy in cases of trilaminar pattern


Factors (n=220)	OR	95% CI	p^a^

**Age**	2.625	0.835-3.204	0.073
**Hyperechoic endometrium**	0.324	0.127-0.825	0.018
**Number of contractions**	2.163	1.180-2.224	0.023
**Weak endometrial movement**	3.251	1.008-1.483	0.048
**Cervicofundal movement**	1.187	0.383-3.680	0.767


^a^; Logistic regression analysis.

## Discussion

The study showed that higher number of contraction and weak or moderate endometrial movement in IUI day is preferable for success of pregnancy. The direction of movement was also better for the pregnancy in cervicofundal contraction consistent with sperm transport. Endometrial thickness and volume were not essential factor for pregnancy; however, endometrial morphology with trilaminar and hypoechoic pattern was more important for pregnancy achievement in IUI cycle stimulated with CC. The effect of endometrial movement was more certain in women with endometrial trilaminar pattern rather than age.

Intrauterine insemination is usually the first line of treatment offered to patients with unexplained infertility, and it is impossible to completely standardize all IUI factors. In one study, a high number of preovulatory follicles, a high motile spermatozoa count (>20×10^6^) before sperm preparation, younger female age (<25 years), a history of secondary infertility and a high percentage of processed sperm with normal morphology (>4%) were associated with the highest pregnancy rate in IUI cycles (12). Ultrasonographic endometrial characteristics, including endometrial thickness, volume, texture, and pelvic flow have been also implicated as crucial factors for the achievement of pregnancy (4, 13).

An additional parameter, endometrial wave-like movement, has been studied by ultrasound in spontaneous, IUI, and IVF cycles (7-9, 14, 15). Endometrial movements originate from subendometrial myometrium (i.e., so-called "junctional zone"), a more compact structure than the rest of myometrium that generates wave-like or peristaltic contractions (16-18). The Junctional zone and the endometrium exhibit cyclic changes in estrogen and progesterone receptor expression. Therefore, junctional zone contractions vary throughout the cycle according to hormonal changes (19). However, the reasons for directional variation between cycles remain unknown.

In general, the endometrium has a baseline activity of random movements that originate from various foci without obvious coordination throughout the menstrual cycle. The random contractions become several distinct types at mid-cycle, together with an increase in the presence of activity that is likely due to elevated estrogen levels (20). The most prominent type of contraction is cervix-to-fundus, as observed in our study, which facilitates sperm transport through the female genital tract (21). Consistent with this hypothesis, human sperms have been detected in the fallopian tubes in a few minutes after vaginal placement (1). During the luteal phase, subendometrial activity decreases under the influence of progesterone, and the women experience short and asymmetrical myometrial contraction waves that often move in opposing directions. These reduced and irregular activities may help the blastocyst implantation near the fundus (i.e., the region of relative peristaltic quiescence) and facilitate a local supply of nutrients and oxygen (22, 23). During the menstrual period, subendometrial contractility is predominantly antegrade, moving from the fundus to the cervix (16). This pattern of activity promotes hemostasis and the forward emptying of uterine content (e.g., menstrual blood) (24).

Subendometrial contractions follow similar patterns, but are more exaggerated throughout stimulated cycles than during the natural cycles. Higher subendometrial contractions and correspondingly increases in the mobility of the endometrium may be unfavorable for the early phase implantation (25). Junctional zone contractions and endometrial wave-like movements in the adjacent endometrium may account for several clinical observations, such as the lower success rate after a difficult embryo transfer or the higher rates of ectopic and heterotopic pregnancies rates after IVF-embryo transfer. For this reason, there is a need for a method of atraumatic embryo transfer that avoids touching the uterine fundus and limits manipulation within the cervical area (26).

The effects of endometrial movement on pregnancy rate in IUI cycles have not been studied. To minimize bias and to maximize the control of variables, we focused on couples with unexplained infertility whose first IUI cycles were stimulated by CC. The direction of contractions was classified according to the two opposite directions that generate endometrial movements, which are supposed to influence sperm transport and implantation. Other types of weak contractions (i.e., transverse, random, or focal activity) were not considered because they exerted little influence on the endometrial stream.

All components of endometrial movements act as mechanical factors in the establishment of pregnancy. It has been suggested that cervicofundal movements, weak or moderate movements, and more than four movements per minute may increase the likelihood of pregnancy during IUI cycles. However, after adjusting for other factors, the direction of subendometrial contractions may be less important in IUI cycles than in natural cycles (20). It is hypothesized that the introduction of sperm into the upper portion of the uterine cavity may overcome disadvantageous endometrial movements, unlike natural cycles. Nevertheless, the pregnancy rate was lower in the fundocervical contraction group than in the cervicofundal group.

Our results showed that pregnancy rates were significantly higher in the presence of weak and moderate endometrial movements than in the presence of strong movements. With the proper number of contractions, active and calm movements can help achieve pregnancy in IUI cycles. According to this hypothesis, a high velocity of contractions facilitates the transport of sperm during IUI procedures (21), and a mild-to-moderate degree of movement would provide an adequate environment for implantation.

Women with endometrial hyperechoic group exhibited weaker and less frequent contractions of the subendometrium, and these factors were associated with a lower pregnancy rate. As an independent factor, or as a contractility-related factor, early hyperechoic changes induced by progesterone indicates premature luteinization and a lower probability of pregnancy (4). Premature luteinization of follicles, which was recently studied with gonadotropin-releasing hormone (GnRH) antagonists in IUI cycles, may cause unexpected endometrial luteolysis and can alter the implantation window (27).

Several conflicting reports have been made regarding the relationship between endometrial thickness/volume and pregnancy rate (4, 9, 13, 28-32). Some groups have reported that an increased endometrial thickness or volume is associated with a higher pregnancy rate (28, 29). However, others have observed no significant association between endometrial thickness/volume and pregnancy outcome (9, 31, 32). In this study, endometrial thickness and volume did not significantly affect pregnancy rate; however, no pregnancies occurred when the endometrial thickness was less than 6 mm or the endometrial volume was less than 2.7 mL. The presence of a trilaminar pattern, a widely utilized marker for uterine receptivity, was also evaluated. Hock et al. demonstrated that the trilaminar pattern was associated with a significantly higher pregnancy rate in IUI patients (33). Other findings have supported this opinion by suggesting that the endometrial pattern provides information for predicting pregnancy rates, with a trilaminar endometrium favorable for further fertility (13).

This study is the first suggest of the relationship between endometrial movements and pregnancy in IUI cycles, which may provide physicians with additive useful information about endometrium when they perform IUI. The endometrial movement in the ovulation period was favorable for the successful pregnancy as previous studies (34). And we also analyzed various endometrial characteristics like endometrial thickness, volume, pattern, and echoicity which have been presumed to be associated with pregnancy achievement. But the limitation of the study is that we analyzed only in patients stimulated by CC, and we did not consider other directions of endometrial movements. Restricted day of the examination may result in the inconsistent outcomes compared with previous researches (35). Further research must be necessary in this area.

## Conclusion

Endometrial mechanical factors and architecture such as hyperechoic change and trilaminar pattern were more important than endometrial thickness and volume in the prediction of pregnancy during an initial IUI cycles stimulated by CC. As endometrial mechanical factors, movement with cervicofundal direction, movements of weak or moderate intensity, and higher number of endometrial contractions per minute should be considered favorable predictors of pregnancy. In patients with endometrial characteristics of a distinct trilaminar pattern, these endometrial contractions can be predictable for the occurrence of pregnancy in IUI stimulated by CC, except the direction of contraction.
